# Species delimitation in the Grayling genus *Pseudochazara* (Lepidoptera, Nymphalidae, Satyrinae) supported by DNA barcodes

**DOI:** 10.3897/zookeys.600.7798

**Published:** 2016-06-22

**Authors:** Rudi Verovnik, Martin Wiemers

**Affiliations:** 1 Department of Biology, Biotechnical Faculty, University of Ljubljana, Jamnikarjev 101, Ljubljana, Slovenia; 2 UFZ – Helmholtz-Centre for Environmental Research, Department of Community Ecology, Theodor-Lieser-Str. 4, 06120 Halle, Germany

**Keywords:** Papilionoidea, Satyrinae, butterflies, phylogeny, barcoding, taxonomy

## Abstract

The Palaearctic Grayling genus *Pseudochazara* encompasses a number of petrophilous butterfly species, most of which are local endemics especially in their centre of radiation in SW Asia and the Balkans. Due to a lack of consistent morphological characters, coupled with habitat induced variability, their taxonomy is poorly understood and species delimitation is hampered. We employed a DNA barcoding approach to address the question of separate species status for several European taxa and provide first insight into the phylogeny of the genus. Unexpectedly we found conflicting patterns with deep divergences between presumably conspecific taxa and lack of divergence among well-defined species. We propose separate species status for *Pseudochazara tisiphone*, *Pseudochazara amalthea*, *Pseudochazara amymone*, and *Pseudochazara kermana* all of which have separate well supported clades, with the majority of them becoming local endemics. Lack of resolution in the ‘Mamurra’ species group with well-defined species (in terms of wing pattern and coloration) such as *Pseudochazara geyeri*, *Pseudochazara daghestana* and *Pseudochazara alpina* should be further explored using nuclear molecular markers with higher genetic resolution.

## Introduction

Depending on which systematic order of classification is adhered to, the genus *Pseudochazara* comprises 27–32 species of Graylings (Gross 1978, Lukhtanov 2007, Savela 2015). It has a wide distribution in the Palaearctic region from North Africa to the Himalayas and Mongolia (Tennent 1996, Tshikolovets 2005, Yakovlev 2012). In addition to vague species delimitation, large intraspecific variation has resulted in the description of over 100 subspecific taxa (Lukhtanov 2007) in this intensively studied taxon.

The main reason for the extensive variation in phenotype can be linked with the specific ecological requirements of these butterflies. They are mostly petrophilous and limited to specific rock substrate to which they are perfectly adapted with their camouflaged underside wing pattern and cryptic coloration. Local adaptation to mimic the coloration of the rock substrate is, therefore, one of the main drivers for such large scale diversification (Lorković 1974, Weiss 1980, Hesselbarth et al. 1995, Tennent 1996, but see Anastassiu et al. 2009).

Trying to resolve the systematics of this genus and its species delimitation has been thwarted by the fact that the genitalia of many *Pseudochazara* species are virtually identical and their wing shape and coloration, both being partially dependant on environmental conditions (Gross 1978, Hesselbarth et al. 1995), is inconsistent. The last comprehensive taxonomic review which was published by Gross (1978) is already outdated. He recognised 24 species, among which *Pseudochazara obscura* (Staudinger, 1878) is now considered a subspecies of *Pseudochazara lydia* (Staudinger, 1878) (see Eckweiler and Rose 1988), *Pseudochazara aurantiaca* (Staudinger, 1878) and *Pseudochazara xerxes* Gross & Ebert, 1975 have been reclassified as subspecies of *Pseudochazara beroe* (Herrich-Schäffer, 1844) (see Lukhtanov 2007), *Pseudochazara schahrudensis* (Staudinger, 1881) is now considered conspecific with *Pseudochazara mamurra* (Herrich-Schäffer, 1844) (see Eckweiler 2004) and *Pseudochazara pakistana* Gross, 1978 is conspecific with either *Pseudochazara gilgitica* (Tytler, 1926) (see Lukhtanov 2007) or *Pseudochazara baldiva* (Moore, 1865) (see Wakeham-Dawson et al. 2007). Several members of the *Pseudochazara* genus from Central Asia that are currently recognised as separate species were considered subspecific taxa in the revision (e.g. *Pseudochazara droshica* (Tytler, 1926), *Pseudochazara gilgitica* (Tytler, 1926), *Pseudochazara lehana* (Moore, 1878)) while *Pseudochazara euxina* (Kuznetsov, 1909) from Crimea was entirely neglected. Two additional species were described after the revision, *Pseudochazara kanishka* (Aussem 1980a) and *Pseudochazara annieae* (Pagès 2007). Following Gross’ revision (1978) the shape of the androconial scales of several *Pseudochazara* species has proven to be constant, enabling species delimitation (Weiss 1980, Eckweiler and Rose 1989, Wakeham-Dawson and Kudrna 2000, Wakeham-Dawson et al. 2003, Wakeham-Dawson and Kudrna 2005, Wakeham-Dawson 2006, Wakeham-Dawson and Kudrna 2006, Pages 2007, Wakeham-Dawson et al. 2007).

There has been no attempt to reconstruct the phylogeny of the genus or validate species status using molecular markers. Only the taxonomic position within subtribe Satyrina and a sister relationship to *Chazara* has been established (Peña et al. 2011).

In order to resolve the relationship among *Pseudochazara* species and re-evaluate their species status, in particular of some European taxa, we employed DNA barcoding – using a standardized gene region (5’ segment of the mitochondrial gene cytochrome *c* oxidase subunit I = COI) which enabled us to utilize additional *Pseudochazara* sequences available in the Barcode of Life Database (BOLD 2015). DNA barcodes have been widely and successfully used in Lepidoptera taxonomy and species delimitation as an additional set of characters which are independent of habitat conditions (Hebert et al. 2004, Nazari and Sperling 2007, Nazari et al. 2010, Dinca et al. 2011, Yang et al. 2012, Lukhtanov and Novikova 2015, Pazhenkova et al. 2015). However, there are several limitations of this method (see e.g. Wiemers and Fiedler 2004, Brower 2006, Ritter et al. 2013, Song et al. 2008, Toews and Brelsford 2012) which should be taken into account in the interpretation of the gene tree.

## Material and methods

### Sample collection, DNA extraction, amplification, sequencing, and alignment

With the aim of achieving consistency, we adopt the nomenclature of the most recent list of *Pseudochazara* species by Lukhtanov (2007). Following the discovery of *Pseudochazara mamurra amymone* in Albania (Eckweiler 2012), we initially sampled all the *Pseudochazara* taxa from the Balkan Peninsula, a hotspot of *Pseudochazara* diversity in Europe (Verovnik et al. 2014, Gascoigne-Pees et al. 2014). We then broadened the range of our sampling adding additional species from Turkey and the Middle East, the main areas of *Pseudochazara* diversification. Altogether 27 specimens belonging to 10 species of *Pseudochazara*, for which the barcoding gene COI was successfully amplified, were included in the study (see Appendix 1). All specimens were dried prior to DNA extraction. In addition, we included COI sequences from 81 individuals belonging to 14 species from the BOLD database (BOLD 2015). Only specimens that could be unambiguously identified by the voucher photos were selected. Following the nomenclature guidelines proposed by Lukhtanov (2007) a total of 34 taxa belonging to 20 species were included in the analysis. As outgroups, we added several sequences of the closely related Satyrine genus *Chazara* from GenBank, based on the results of the phylogenetic study of Satyrinae by Peña et al. (2011).

Total genomic DNA was extracted from single legs, following the Mammalian tissue preparation protocol (GenElute Mammalian Genomic DNA miniprep kit from Sigma-Aldrich). For each sample a 657 bp fragment of the first subunit of the mitochondrial gene cytochrome *c* oxidase (COI) was amplified using primers LCO1490 and HCO2198 (Folmer et al. 1994). Amplification followed a standard protocol described in Verovnik et al. (2004). PCR products were visualized on an agarose gel to verify amplification success and sequenced by Macrogen in both directions on an Applied Biosystems 3730xl sequencer.

### Phylogenetic analysis

We used Bayesian inference to reconstruct a phylogenetic tree. To achieve more clarity the tree was constructed on a subset of samples including only unique haplotypes belonging to the same taxon. A hierarchical likelihood test was employed in order to test alternative models of evolution, using JModeltest v.0.1.1 (Posada 2008). A GTR (Generalised time reversible) model of nucleotide substitution with gamma distributed rate heterogeneity and a significant proportion of invariable sites was selected in accordance with the Akaike Information Criterion. Bayesian analysis was performed with MrBayes v.3.1.2 implementing the best fit substitution model (Huelsenbeck and Ronquist 2001). Markov chain Monte Carlo search was run with four chains for 4 × 10^6^ generations, taking samples every 100 generations. The approximate number of generations needed to obtain stationarity of the likelihood values (‘‘burn-in’’) of the sampled trees was estimated graphically to 2000 trees. From the remaining trees posterior probabilities were assessed for individual clades based on their observed frequencies. Trees were visualised using Figtree v.1.4.2 (Rambaut 2014). Genetic distances (p-) were calculated with MEGA 6.0 (Tamura et al. 2013). In addition, a statistical parsimony network analysis was performed with TCS 1.21 (Clement et al. 2000).

## Results

**Figure 1.**
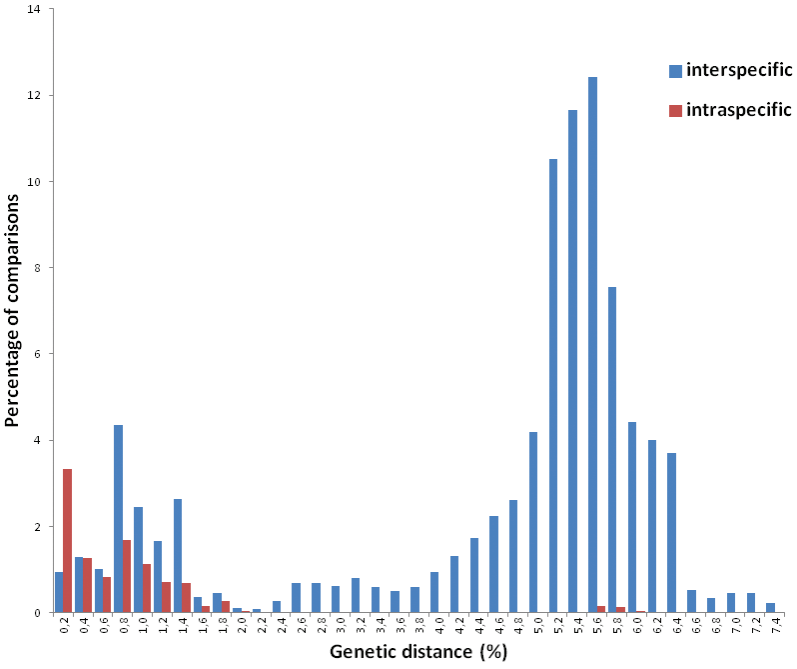
Frequency distribution of pairwise intra- and interspecific p-distances of the COI sequences in the genus *Pseudochazara*. No “barcoding gap” exists between these two data series.

No insertions or deletions were observed in the mitochondrial COI gene and therefore the alignment was unambiguous. For the COI dataset 63 unique haplotypes among 108 *Pseudochazara* sequences were detected. 114 (17.5%) sites were variable and 95 (14.6%) were parsimony informative. The average interspecific genetic distance was 4.9%, but in the case of *Pseudochazara mniszechii* the intraspecific diversity ranged from 0 to 6.7% with highly distinct divergent sequences of *Pseudochazara mniszechii tisiphone*. No evident barcoding gap was observed separating intraspecific from interspecific pairwise genetic distances (Fig. 1). On the contrary, sharing of identical haplotypes was observed in the following taxa: *Pseudochazara graeca* / *Pseudochazara mamurra amymone*, *Pseudochazara mamurra mamurra* / *Pseudochazara daghestana*, and *Pseudochazara beroe aurantiaca* / *Pseudochazara alpina*. On the other hand, 82% of species comparisons showed high (≥2%) interspecific distances.

**Figure 2.**
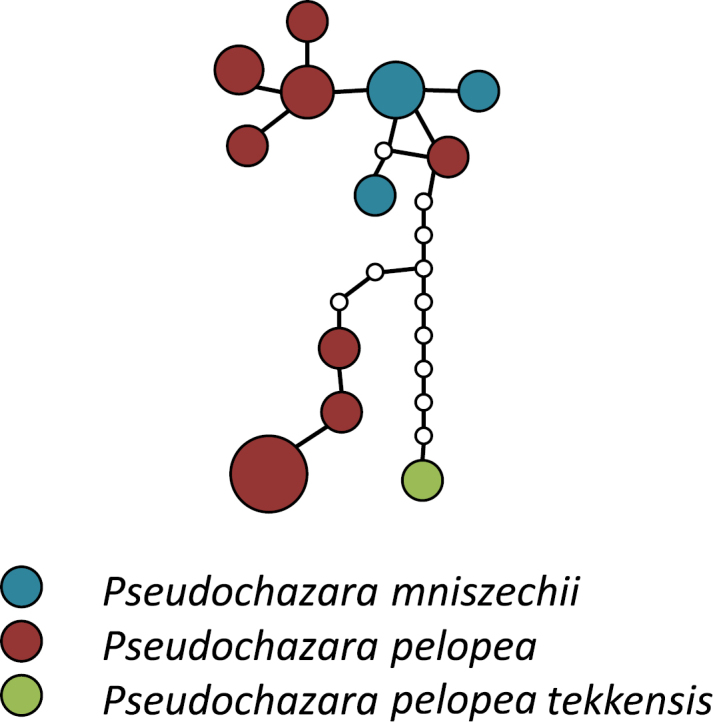
Statistical Parsimony network of the ‘pelopea’ species group. Coloured circles represent COI haplotypes and their size corresponds to the number of samples per haplotype. Small white circles represent unsampled haplotypes.

**Figure 3.**
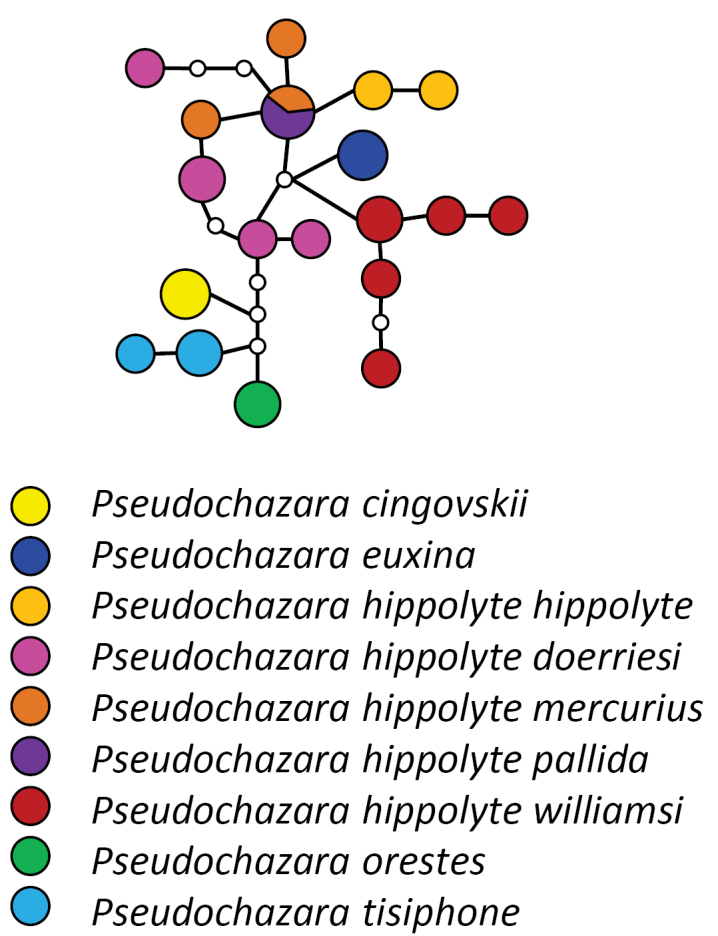
Statistical Parsimony network of the ‘hippolyte’ species group. Coloured circles represent COI haplotypes and their size corresponds to the number of samples per haplotype. Small white circles represent unsampled haplotypes.

**Figure 4.**
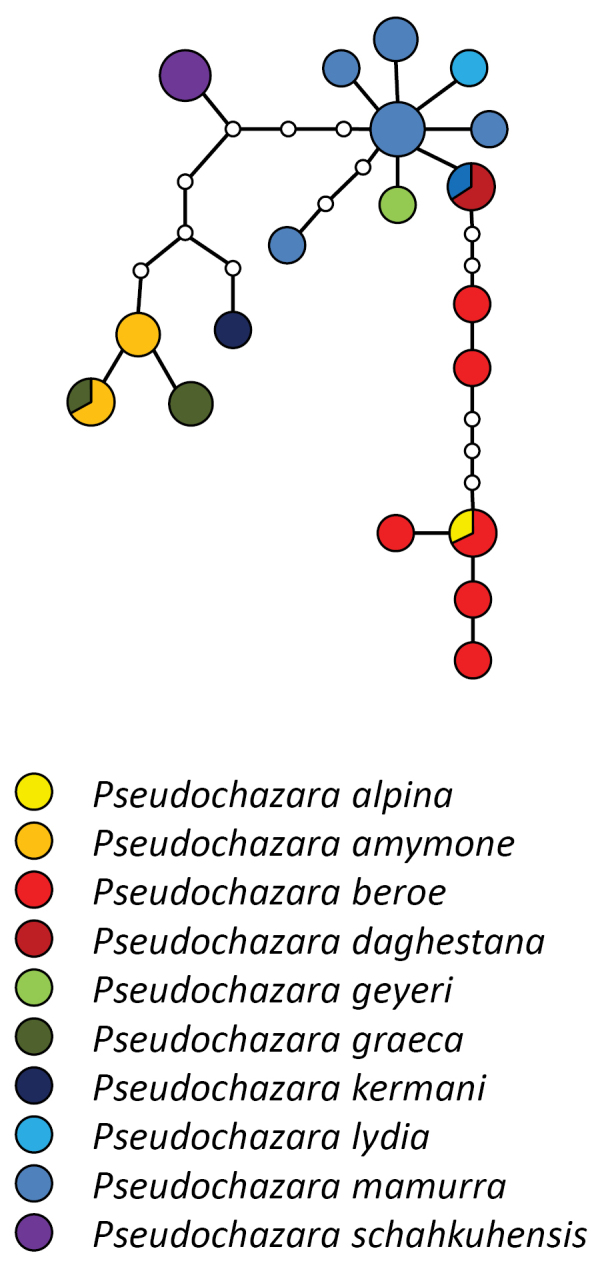
Statistical Parsimony network of the ‘mamurra’ species group. Coloured circles represent COI haplotypes and their size corresponds to the number of samples per haplotype. Small white circles represent unsampled haplotypes.

The calculated maximum connection for parsimony networks at the default 95% limit was 11 steps, and resulted in 9 separate networks within *Pseudochazara*. 6 of them contain only single species (*Pseudochazara atlantis*, *Pseudochazara turkestana*, *Pseudochazara thelephassa*, *Pseudochazara lehana*, *Pseudochazara kanishka*, and *Pseudochazara anthelea*), whereas the remaining 3 comprise several closely related species (Figs 2–4). Outgroups were contained in 2 distinct networks (*Chazara enervata* and *Chazara briseis*/*Chazara heydenreichi*).

**Figure 5.**
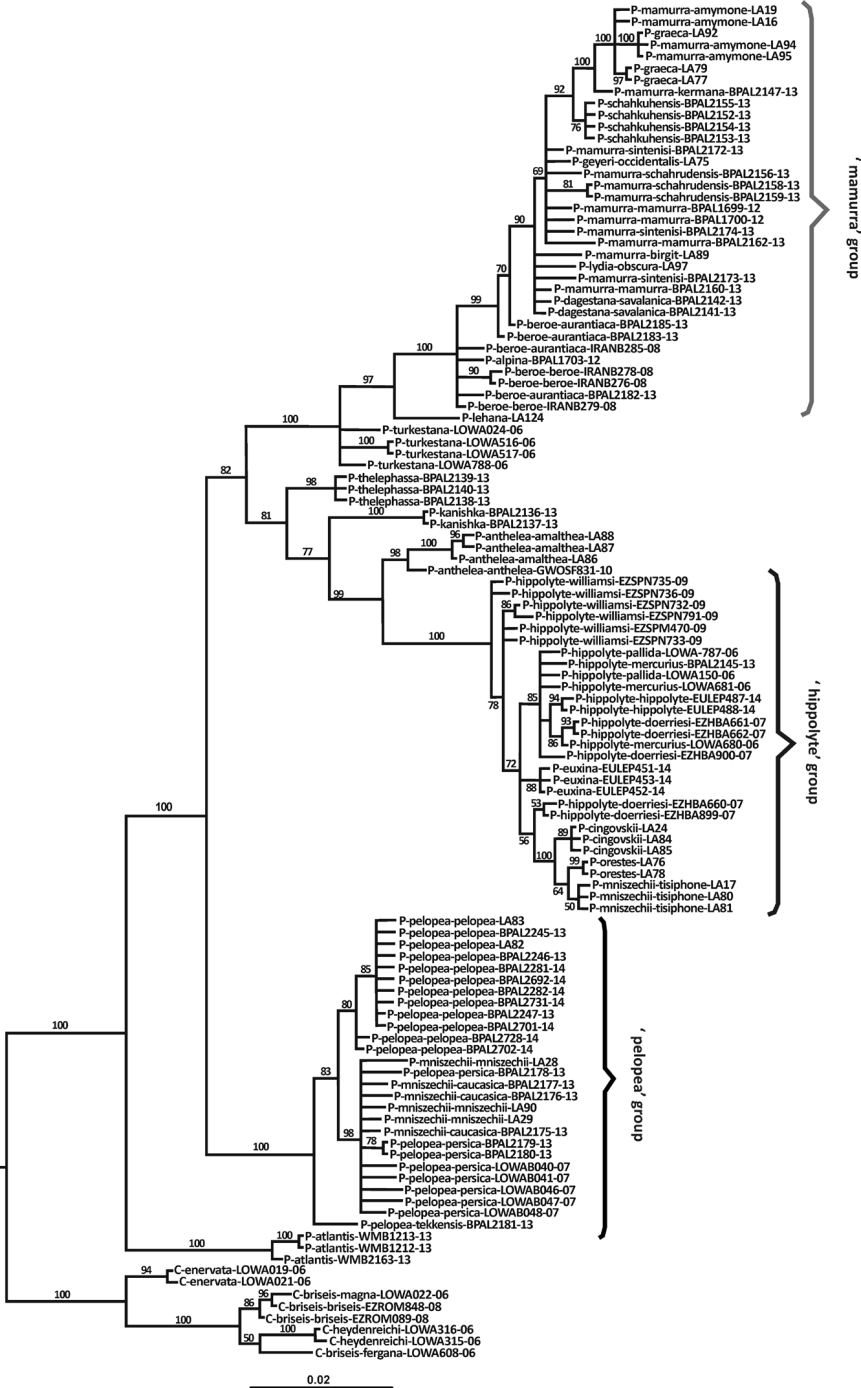
Phylogeny of *Pseudochazara* species derived from the barcoding gene COI using Bayesian inference analysis. Values on major branches are Bayesian posterior probabilities. Branches with support lower than 50% were collapsed manually. Branch names combine taxon name and sample ID (see Appendix 1). Nomenclature follows Lukhtanov (2007).

The topology of the Bayesian Inference tree of all *Pseudochazara* samples, including the selected outgroup species (Fig. 5), confirms the monophyly of the genus. High posterior probability values support a basal position of *Pseudochazara atlantis*, the only species of the genus present in (and confined to) North Africa. This is somewhat surprising as *Pseudochazara anthelea* and *Pseudochazara thelephassa* are considered to be morphologically the most distinct and separate species within the genus (Gross 1978). *Pseudochazara atlantis* has tentatively been placed into two groups, the ‘*mamurra*’ species group (Brown 1976), based on androconia shape, and the ‘*pelopea*’ species group (Wakeham-Dawson and Dennis 2001), on account of the shape of male genitalia. *Pseudochazara atlantis* is also distinctive according to the TCS analysis and forms a separate network. In addition, the second basal split within *Pseudochazara* is well supported, and, apart from some single species clades, three species groups tentatively named as the ‘*pelopea*’, ‘*hippolyte*’ and ‘*mamurra*’ clades received high support. We present the results for these clades separately:

### ‘*Pelopea*’ group

This group, which forms a distinct network in the TCS analysis (Fig. 2), includes two species, *Pseudochazara pelopea* and *Pseudochazara mniszechii*. However, there is no genetic differentiation between them, with *Pseudochazara pelopea persica* and *Pseudochazara pelopea caucasica* intermixed with *Pseudochazara mniszechii*. Two well supported clades pertain to geographically isolated subspecies of *Pseudochazara pelopea*, the Levant region (nominotypic *Pseudochazara pelopea pelopea*) and Kopet Dhag in NE Iran (*Pseudochazara pelopea tekkensis*). Both subspecies are morphologically distinct from *Pseudochazara pelopea persica*, in particular the latter, with much wider and more pronounced orange submarginal bands on their forewings. *Pseudochazara pelopea tekkensis* is considered a separate species by Nazari (2003). *Pseudochazara mniszechii* is also polyphyletic due to the separate position of the subspecies *tisiphone* from the southern Balkans, which is clearly not closely related, and belongs to the ‘*hippolyte*’ group.

### ‘*Hippolyte*’ group

The ‘*hippolyte*’ clade *sensu stricto* includes the widely distributed *Pseudochazara hippolyte* complex which has a vast range from southern Spain to central China (Tshikolovets 2011) together with a number of local endemics from the southern Balkan Peninsula: *Pseudochazara cingovskii* in the Republic of Macedonia, *Pseudochazara orestes* from north-eastern Greece and the neighbouring part of Bulgaria, *Pseudochazara mniszechii tisiphone* from north-western Greece and southern Albania and *Pseudochazara euxina* from the Crimean Peninsula. Both, the haplotype network analysis (Fig. 3) and the phylogeny (Fig. 5) show that *Pseudochazara mniszechii tisiphone* is not a subspecies of *Pseudochazara mniszechii* despite superficial resemblance in wing patterns and coloration. In fact, it is closely related to two other local endemics from the Balkan Peninsula, *Pseudochazara cingovskii* and *Pseudochazara orestes*. The presence of *Pseudochazara mniszechii tisiphone* in the western part of Turkey, near Bursa (Hesselbarth et al. 1995) remains to be verified. The single haplotype of *Pseudochazara euxina* is nestled among samples of *Pseudochazara hippolyte*, so our preliminary results do not support its current status as a separate species. Within this clade *Pseudochazara hippolyte williamsi* from southern Spain appears basally, however with low posterior probability and it is not monophyletic. All other described subspecies (*Pseudochazara hippolyte pallida*, *Pseudochazara hippolyte doerriesi*, *Pseudochazara hippolyte mercurius*) are less distinct from the nominotypical subspecies, with two Central Asiatic subspecies (*Pseudochazara hippolyte pallida*, *Pseudochazara hippolyte mercurius*) sharing haplotypes.

The sister relationship of *Pseudochazara thelephassa* and *Pseudochazara anthelea*, which is indicated by genital morphology (the presence of a distinct costal process on the dorsal side of the valve) and wing pattern (the presence of a well-defined black area in the forewing discal cell) (Aussem 1980b, Hesselbarth et al. 1995, Wakeham-Dawson and Dennis 2001), could not be corroborated as *Pseudochazara anthelea* appears to be a sister clade to the ‘*hippolyte*’ group *sensu strictu* with high posterior probability. *Pseudochazara kanishka* from Tajikistan is a sister species of the *anthelea*-*hippolyte* clade, while *Pseudochazara thelephassa* is sister taxon to the *anthelea*-*hippolyte*-*kanishka* clade, however, with low support. These results concur with wing pattern, i.e. a well-defined black area in the forewing discal cell, also present in specimens of *Pseudochazara kanishka*.

It is important to note that the average genetic distance between two geographically separated subspecies, *Pseudochazara anthelea anthelea* from Asia Minor and neighbouring islands, and *Pseudochazara anthelea amalthea* from the Balkan Peninsula was 1.5%. This result is indicative for differentiation into distinct species as predicted by Kudrna et al. (2011).

In the TCS analysis, this group is split into 3 networks: a) the *hippolyte* clade *sensu stricto* (Fig. 3), b) *Pseudochazara anthelea*, and c) *Pseudochazara thelephassa*.

### ‘*Mamurra*’ group

The only two entirely Central Asian species available for analysis, *Pseudochazara turkestana* and *Pseudochazara lehana*, form a well-supported clade together with the ‘*mamurra*’ group, indicating their close relationship, but with a separate network for each in the TCS analysis. All other sequences form a single network (Fig. 4). Although the species sampling in Central Asia is incomplete, there is no evidence of a deep split between Asiatic and European/African taxa as predicted by Wakeham-Dawson and Dennis (2001). The ‘*mamurra*’ group is monophyletic, and includes several well-defined species (in terms of wing patterns, androconia and genitalia) with identical or very similar haplotypes. The following taxa could not be distinguished based on COI haplotypes as they do not form separate monophyletic clades: *Pseudochazara mamurra*, *Pseudochazara beroe*, *Pseudochazara geyeri*, *Pseudochazara daghestana*, *Pseudochazara alpina*, and *Pseudochazara lydia*. Only a single sequence was obtained for *Pseudochazara geyeri* and *Pseudochazara lydia*, so their position within this group is tentative. However, it is clear that *Pseudochazara lydia* is closely related to *Pseudochazara mamurra* with which it shares similarities e.g. the shape of the androconia (Wakeham-Dawson 2005). *Pseudochazara alpina* shares the haplotype with *Pseudochazara beroe* and they appear closely related, however, this is again based on the inclusion of a single sequence.

Within the ‘*mamurra*’ group the only well supported clade includes the taxa *Pseudochazara schahkuhensis*, *Pseudochazara mamurra kermana*, *Pseudochazara graeca* and *Pseudochazara mamurra amymone*. While *Pseudochazara schahkuhensis* is sympatric in part of its range with *Pseudochazara mamurra*, all other taxa have geographically isolated ranges. *Pseudochazara graeca* and *Pseudochazara mamurra amymone* are present in the southern part of the Balkan Peninsula with partial range overlap (Pamperis 2009). Both species are clearly morphologically distinct, but genetically not identifiable in COI haplotypes. Clearly this relationship puts in question the status of *Pseudochazara mamurra amymone* as a subspecies of *Pseudochazara mamurra*. The same conclusion can be drawn for *Pseudochazara mamurra kermana* from Iran (Kerman province), which is also well placed within this clade as a sister species to both southern Balkan Peninsula taxa.

## Discussion

Our study supports the monophyly of the genus *Pseudochazara* with high posterior probability values of the COI gene tree. Within the genus, however, two conflicting patterns appear with, unexpectedly, deep divergences between presumably conspecific taxa on the one hand and lack of divergence among well-defined species on the other. This is to some extent concordant with similar studies in related genera in the subfamily Satyrinae (Kodandaramaiah and Wahlberg 2009, Nazari et al. 2010, Kreuzinger et al. 2014). The basal position of *Pseudochazara atlantis* from North-western Africa as sister group to all remaining *Pseudochazara* species falls into the first category. Based on distinct male genitalia morphology and wing shape/patterns *Pseudochazara anthelea* and *Pseudochazara thelephassa* were considered to form the basal split within the genus (Gross 1978, Aussem 1980b, Hesselbarth et al. 1995, Wakeham-Dawson and Dennis 2001). The basal position of *Pseudochazara atlantis* is difficult to explain in terms of biogeography, as it indicates a North African origin of the genus, which has its centre of divergence much further eastwards in the Middle East (Hesselbarth et al. 1995, Tshikolovets 2011). *Pseudochazara atlantis* is an alpine species distributed only in the Atlas Mountains of Morocco (Tennent 1996), therefore its isolation from the main distribution of the genus could possibly have preceded the last land bridge connections with Europe at the end of the Miocene (Garcia-Castellanos et al. 2009). Hence, its basal position could be an artefact of long-branch attraction (Bergsten 2005) and/or incomplete sampling of the entirely Asiatic species. Therefore, confirmation with additional genetic markers and additional sampling is required.

Another unexpected result is a deep split between *Pseudochazara mniszechii* and *Pseudochazara mniszechii tisiphone*, species which are very similar in wing patterns/coloration and considered conspecific in current literature (Hesselbarth et al. 1995, Kudrna et al. 2011, Tshikolovets 2011, Eckweiler 2012) and databases (Lukhtanov 2007, Savela 2015, Fauna Europaea 2016). Based on the COI gene tree *Pseudochazara tisiphone* Brown, 1980 (stat. n.) is a separate species closely related to two local endemics from the southern part of the Balkan Peninsula, *Pseudochazara orestes* and *Pseudochazara cingovskii*. Actually *Pseudochazara tisiphone* was originally described as a subspecies of *Pseudochazara cingovskii* (Brown 1980) and its close relationship was hypothesised also by Wakeham-Dawson and Dennis (2001) based on the similarity of the male genitalia. The low level of genetic differentiation between *Pseudochazara tisiphone*, *Pseudochazara orestes*, and *Pseudochazara cingovskii* indicates a relatively recent speciation, however, we are inclined towards supporting their separate species status based on constant differences in wing patterns/coloration and also their ecological specialization (Pamperis 2009, Verovnik et al. 2013).

A split between *Pseudochazara anthelea anthelea* from Asia Minor and *Pseudochazara anthelea amalthea* from the Balkan Peninsula has been suggested based on minor differences in male genitalia and consistent differences in female wing coloration between both taxa (Olivier 1996, Wakeham-Dawson and Dennis 2001). They are considered separate morphospecies by Kudrna et al. (2011). We can agree with separate species status as the split between the two taxa is much older compared to almost no differentiation in three morphologically and ecologically well defined species: *Pseudochazara tisiphone*, *Pseudochazara orestes*, and *Pseudochazara cingovskii*. Following this reasoning, *Pseudochazara pelopea tekkensis* from NE Iran could also be considered a distinct species, however, inclusion of more samples is needed to confirm this status.

Given the high resolution of the basal clades within the COI gene tree, the lack of differentiation between taxa within the ‘*mamurra*’ and ‘*pelopea*’ group was unexpected. In particular, species like *Pseudochazara geyeri* and *Pseudochazara daghestana* are among the most easily recognisable species in the genus with uniform and very distinct wing patterns/coloration. There are several possible hypotheses to explain this lack of differentiation:

– Incomplete lineage sorting: recent speciation could result in unresolved relationships among these closely related species; however, well-defined species borders in terms of constant wing pattern differentiation coupled with broad overlaps in species ranges challenges this hypothesis.

– Recent gene flow: gene flow between closely related taxa is a known phenomenon (Descimon and Mallet 2009) and masks relationships among species especially with mitochondrial DNA (Gompert et al. 2008). The species involved have broadly overlapping ranges and could sometimes be found syntopic (Aussem 1980c, Hesselbarth et al. 1995), so hybridization is possible. Actually hybridization is documented even among the most distantly related species such as *Pseudochazara anthelea* and *Pseudochazara geyeri* (Aussem, 1980c). Nuclear markers with higher genetic resolution (e.g. microsatellites, SNPs) would be required to study the contact zones between these taxa to confirm ongoing gene flow. It must be noted that partial exclusion is evident when two or more *Pseudochazara* species are syntopic, as one is always dominant, while the others appear in very low frequencies (Hesselbarth et al. 1995, Verovnik et al. 2014).

– Pseudogenes or *Wolbachia* infections: both are common in invertebrates, particularly in arthropods (Bensasson et al. 2011, Gerth et al. 2014, Leite 2012, Ritter et al. 2013). As the vast majority of the haplotypes in the ‘*mamurra*’ and ‘*pelopea*’ clades originate from the BOLD database it is impossible to check or correct for this potential error.

The most enigmatic taxon among the '*mamurra*' group is *Pseudochazara mamurra amymone* from northern Greece and Albania (Eckweiler 2012, Verovnik et al. 2014). Apart from the author’s original description (Brown 1976) little has been published regarding this elusive taxon for a long time. Failed attempts to locate the vaguely described type locality (Cuvelier 2010) have led to several misleading hypotheses, resulting in speculation that it may even be a rare hybrid between *Pseudochazara tisiphone* and *Pseudochazara anthelea* (Wakeham-Dawson and Dennis 2001, Kudrna et al. 2011). Somewhat surprisingly, the COI gene tree suggests it has a close relationship with *Pseudochazara graeca*, another species from the southern Balkan Peninsula. These two taxa have distinct and constant wing patterns and differ in their habitat requirements, with *Pseudochazara mamurra amymone* inhabiting steep and hot rocky gorges at lower elevations (Gascoigne-Pees et al. 2014) while *Pseudochazara graeca* is predominantly a montane (high elevation) species endemic to Greece (Anastassiu et al. 2009). Thus, despite paraphyly of *Pseudochazara amymone* Brown, 1976 (stat. n.) in relation to *Pseudochazara graeca*, we believe they both represent valid species within the ‘*mamurra*’ group. Consequently *Pseudochazara kermana* Eckweiler, 2004 (stat. n.), sister species to *Pseudochazara amymone* and *Pseudochazara graeca* combined, should also be elevated to species rank, although additional populations of *Pseudochazara mamurra* in Iran should be examined to confirm this status. Alternatively, all the taxa within the ‘*mamurra*’ group, including the monophyletic *Pseudochazara schakuhensis*, a sister species to the *amymone*-*graeca*-*kermana* clade, should be treated as a single very polymorphic species, a rather more destructive approach given the current taxonomy.

Although we are aware of the pitfalls of using single gene trees in the interpretation of phylogenetic patterns (Nichols 2001), we believe that strongly supported basal branching and splits between taxa, considered conspecific, represent valid insights into speciation in the *Pseudochazara* genus and together with distinct morphology and ecology allows species delimitation. Hence, we propose separate species status for the following taxa: *Pseudochazara tisiphone*, *Pseudochazara amalthea*, *Pseudochazara amymone*, and *Pseudochazara kermana*. This has important conservation implications, as most of these species are local endemics and therefore potentially threatened (Verovnik et al. 2014). Wider taxon sampling and inclusion of nuclear markers would undoubtedly help to a better understanding of the taxonomy of this fascinating butterfly genus.

## Appendix 1

**Table 1.** List of samples of the genus *Pseudochazara* included in the barcoding analysis (either own samples with “LA” ID or from BOLD).

ID	GenBank	Species	Location	Lat	Long	Date	Legit
LA16	KU499958	*Pseudochazara mamurra amymone*	Baboshtice, Körce, Albania	40°31.038'N	20°47.647'E	11.vii.2012	Rudi Verovnik
LA17	KU499959	*Pseudochazara mniszechii tisiphone*	Baboshtice, Körce, Albania	40°31.038'N	20°47.647'E	11.vii.2012	Rudi Verovnik
LA19	KU499960	*Pseudochazara mamurra amymone*	Devoll Gorge, Körce, Albania	40°42.576'N	20°31.446'E	10.vii.2012	Rudi Verovnik
LA24	KU499961	*Pseudochazara cingovskii*	Pletvar Pass, Prilep, Macedonia	41°22.456'N	21°38.805'E	14.vii.2010	Rudi Verovnik
LA28	KU499962	*Pseudochazara mniszechii*	Sivas, Turkey	39°41.519'N	36°59.877'E	22.vii.2009	Tarkan Soyhan
LA29	KU499963	*Pseudochazara mniszechii*	Eskişehir, Turkey	39°43.801'N	30°31.428'E	16.vi.2007	Tarkan Soyhan
LA75	KU499964	*Pseudochazara geyeri occidentalis*	Galičica Pass, Macedonia	40°57.379'N	20°48.961'E	30.vii.2013	Filip Franeta
LA76	KU499965	*Pseudochazara orestes*	Falakro Mt., Greece	41°16.138'N	24°3.947'E	7.vii.2013	Filip Franeta
LA77	KU499966	*Pseudochazara graeca*	Katara Pass, Metsova, Greece	39°47.580'N	21°12.272'E	22.vii.2012	Filip Franeta
LA78	KU499967	*Pseudochazara orestes*	Granitis, Drama,Greece	41°18.533'N	23°54.862'E	27.vii.2013	Rudi Verovnik
LA79	KU499968	*Pseudochazara graeca*	Katara Pass, Metsova, Greece	39°47.580'N	21°12.272'E	26.vii.2013	Rudi Verovnik
LA80	KU499969	*Pseudochazara mniszechii tisiphone*	Drenovë, Korcë, Albania	40°35.352'N	20°48.508'E	21.vii.2013	Rudi Verovnik
LA81	KU499970	*Pseudochazara mniszechii tisiphone*	Drenovë, Korcë, Albania	40°35.352'N	20°48.508'E	21.vii.2013	Rudi Verovnik
LA82	KU499971	*Pseudochazara pelopea*	Mt. Hermon, Israel	33°19.766'N	35°47.243'E	2013	Dubi Benyamini
LA83	KU499972	*Pseudochazara pelopea*	Mt. Hermon, Israel	33°19.766'N	35°47.243'E	2013	Dubi Benyamini
LA84	KU499973	*Pseudochazara cingovskii*	Pletvar Pass, Prilep, Macedonia	41°22.456'N	21°38.805'E	2013	Filip Franeta
LA85	KU499974	*Pseudochazara cingovskii*	Pletvar Pass, Prilep, Macedonia	41°22.456'N	21°38.805'E	2013	Filip Franeta
LA86	KU499975	*Pseudochazara anthelea amalthea*	Veles, Topolka, Macedonia	41°41.915'N	21°46.927'E	2010	Filip Franeta
LA87	KU499976	*Pseudochazara anthelea amalthea*	Mt. Parnassos, Greece	38°31.233'N	22°36.566'E	2010	Filip Franeta
LA88	KU499977	*Pseudochazara anthelea amalthea*	Drenovë, Korcë, Albania	40°35.352'N	20°48.508'E	2013	Filip Franeta
LA89	KU499978	*Pseudochazara mamurra birgit*	Mt. Aladaglar, Turkey	37°47.568'N	35°9.242'E	2006	Filip Franeta
LA90	KU499979	*Pseudochazara mniszechii*	Mt. Aladaglar, Turkey	37°47.568'N	35°9.242'E	2006	Filip Franeta
LA92	KU499980	*Pseudochazara graeca*	Mt. Iti, Greece	38°49.333'N	22°16.635'E	1999	Filip Franeta
LA94	KU499981	*Pseudochazara mamurra amymone*	Drenovë, Korcë, Albania	40°35.352'N	20°48.508'E	2013	Filip Franeta
LA95	KU499982	*Pseudochazara mamurra amymone*	Devoll Gorge, Körce, Albania	40°42.576'N	20°31.446'E	2013	Filip Franeta
LA97	KU499983	*Pseudochazara lydia obscura*	Mersin, Turkey	36°57.017'N	34°23.019'E	12.vii.2010	Tarkan Soyhan
LA124	KU499984	*Pseudochazara lehana*	Saabo Digur La, Ladakh, India	34°10.554'N	77°39.529'E	15.vii.2013	Joseph Verhulst
BPAL1699–12		*Pseudochazara mamurra*	Azerbaijan: near Shamkir, 1300 m	40.6989	45.8697	31.vii.2011	Tikhonov V.
BPAL1700–12		*Pseudochazara mamurra*	Azerbaijan: near Shamkir, 1300 m	40.6989	45.8697	31.vii.2011	Tikhonov V.
BPAL1703–12		*Pseudochazara alpina*	Russia: North Ossetia-Alania, rv. Ardon, Skasan, 1850 m	42.6956	43.9989	12.viii.2011	Tikhonov V.
BPAL2136–13		*Pseudochazara kanishka*	Tajikistan: Khodra-Mumin Mnt.			26.v.2001	A. Petrov
BPAL2137–13		*Pseudochazara kanishka*	Tajikistan: Khodra-Mumin Mnt.			26.v.2001	A. Petrov
BPAL2138–13		*Pseudochazara thelephassa*	Iran: Char Mahall-o-Bahtiyari, Sahr-e-Kord, 2000 m			28.v.2002	P. Hofmann
BPAL2139–13		*Pseudochazara thelephassa*	Iran: Kerman, Kuh-e-Madvar, 5 km S Jowzan, 2400–2600 m			24.v.2002	P. Hofmann
BPAL2140–13		*Pseudochazara thelephassa*	Iran: Kerman, Kuh-e-Segoch, Mahan Pass, 2400–2600 m			21.v.2002	P. Hofmann
BPAL2141–13		*Pseudochazara dagestana savalanica*	Iran: Azarbayjan-e-Sharqi, N Taran, Kuh-e-Sabalan, 2900–3000 m			10.vii.2001	Westphal
BPAL2142–13		*Pseudochazara dagestana savalanica*	Iran: Azarbayjan-e-Sharqi, N Taran, Kuh-e-Sabalan, 2900–3000 m			10.vii.2001	Westphal
BPAL2145–13		*Pseudochazara hippolyte mercurius*	China: Xinjiang, Tian Shan, Borohoro Shan, 40 km SSW Kytun, 1850–2050 m	44.0939	84.7942	08.vii.2006	Grieshuber
BPAL2147–13		*Pseudochazara mamurra kermana*	Iran: Kerman, Kuh-e-Madvar, 5 km S Jowzan, 2200–2400 m			28.v.1999	P. Hofmann
BPAL2152–13		*Pseudochazara schahkuhensis*	Iran: Khorasan, Kopet Dagh, 15 km E Emam Qoli, N Quchan, 2100–2200 m			19.vi.2001	P. Hofmann
BPAL2153–13		*Pseudochazara schahkuhensis*	Iran: Khorasan, Kopet Dagh, Qoucan, 1800 m			13.vii.2000	Hacz-Köszegi
BPAL2154–13		*Pseudochazara schahkuhensis*	Iran: Khorasan, Kopet Dagh, Qoucan, 1800 m			14.vii.2000	Hacz-Köszegi
BPAL2155–13		*Pseudochazara schahkuhensis*	Iran: Khorasan, Kopet Dagh, Qoucan, 1800 m			15.vii.2000	Hacz-Köszegi
BPAL2156–13		*Pseudochazara mamurra schahrudensis*	Iran: Tehran, Elburs, Tuchal, 2400–2600 m			16.vi.2001	P. Hofmann
BPAL2158–13		*Pseudochazara mamurra schahrudensis*	Iran: Tehran, Elburs, Tuchal, 2400–2600 m			16.vi.2001	P. Hofmann
BPAL2159–13		*Pseudochazara mamurra schahrudensis*	Iran: Tehran, Elburs, Tuchal, 2400–2600 m			16.vi.2001	P. Hofmann
BPAL2160–13		*Pseudochazara mamurra mamurra*	Turkey: Artvin, Kilickaya, 1100–1200 m			01.vi.1998	P. Hofmann
BPAL2162–13		*Pseudochazara mamurra mamurra*	Turkey: Erzurum, Dikmen, SW Üzundere, 1300 m			16.vii.1998	P. Hofmann
BPAL2172–13		*Pseudochazara mamurra sintenisi*	Turkey: Bayburt, 5 km N Bayburt, 1500 m			10.vii.1998	P. Hofmann
BPAL2173–13		*Pseudochazara mamurra sintenisi*	Turkey: Erzincan, 5 km SE Caglayan, 1400 m			08.vii.1998	P. Hofmann
BPAL2174–13		*Pseudochazara mamurra sintenisi*	Turkey: Gümüshane, Demirkaynak, 13 km SW Torul, 1100 m			06.vii.1998	P. Hofmann
BPAL2175–13		*Pseudochazara mniszechii caucasica*	Turkey: Bayburt, 5 km N Bayburt, 1500 m			10.vii.1998	P. Hofmann
BPAL2176–13		*Pseudochazara mniszechii caucasica*	Turkey: Erzincan, 5 km SE Caglayan, 1400 m			08.vii.1998	P. Hofmann
BPAL2177–13		*Pseudochazara mniszechii caucasica*	Turkey: Erzurum, road Bayburt-Ispir, Laleli, 1300–1400 m			11.vii.1998	P. Hofmann
BPAL2178–13		*Pseudochazara pelopea persica*	Iran: Char Mahall-o-Bahtiyari, Sahr-e-Kord, 2000 m			28.v.2002	P. Hofmann
BPAL2179–13		*Pseudochazara pelopea persica*	Iran: Kerman, Kuh-e-Madvar, 5 km S Jowzan, 2400–2600 m			24.v.2002	P. Hofmann
BPAL2180–13		*Pseudochazara pelopea persica*	Iran: Kerman, Kuh-e-Madvar, 5 km S Jowzan, 2400–2600 m			24.v.2002	P. Hofmann
BPAL2181–13		*Pseudochazara pelopea tekkensis*	Iran: Khorasan, Kopet Dagh, 15 km E Emam Qoli, N Quchan, 2100–2200 m			19.vi.2001	P. Hofmann
BPAL2182–13		*Pseudochazara beroe aurantiaca*	Iran: Tehran, Elburs, 15 km NE Firuzkuh pass, 1300–2400 m			24.vii.2000	P. Hofmann
BPAL2183–13		*Pseudochazara beroe aurantiaca*	Iran: Mazandaran, Khosh-Yeylaq, 65 km NE Shahrud, 2000–2100 m			23.vi.2001	P. Hofmann
BPAL2185–13		*Pseudochazara beroe aurantiaca*	Iran: Khorasan, Kopet Dagh, 15 km E Emam Qoli, N Quchan, 2100–2200 m			19.vi.2001	P. Hofmann
BPAL2245–13		*Pseudochazara pelopea pelopea*	Israel			22.vi.2013	V.A.Lukhtanov & A.V.Novikova
BPAL2246–13		*Pseudochazara pelopea pelopea*	Israel			22.vi.2013	V.A.Lukhtanov & A.V.Novikova
BPAL2247–13		*Pseudochazara pelopea pelopea*	Israel			22.vi.2013	V.A.Lukhtanov & A.V.Novikova
BPAL2281–14		*Pseudochazara pelopea pelopea*	Syria: Bloudan, 1500 m			16.vii.1999	A, Salk
BPAL2282–14		*Pseudochazara pelopea pelopea*	Syria: Bloudan, 1500 m			16.vii.1999	A, Salk
BPAL2692–14		*Pseudochazara pelopea pelopea*	Israel			03.vii.2014	V.Lukhtanov & A. Novikova
BPAL2701–14		*Pseudochazara pelopea pelopea*	Israel			03.vii.2014	V.Lukhtanov & A. Novikova
BPAL2702–14		*Pseudochazara pelopea pelopea*	Israel			03.vii.2014	V.Lukhtanov & A. Novikova
BPAL2728–14		*Pseudochazara pelopea pelopea*	Israel			04.vii.2014	V.Lukhtanov
BPAL2731–14		*Pseudochazara pelopea pelopea*	Israel			04.vii.2014	V.Lukhtanov
EULEP451–14		*Pseudochazara euxina*	Ukraine			11.vii.2007	local collector
EULEP452–14		*Pseudochazara euxina*	Ukraine			11.vii.2007	local collector
EULEP453–14		*Pseudochazara euxina*	Ukraine			11.vii.2007	local collector
EULEP487–14		*Pseudochazara hippolyte hippolyte*	Russia	52.65	59.5667	23.vii.1998	K. Nupponen
EULEP488–14		*Pseudochazara hippolyte hippolyte*	Russia	51.8	57.0833	14.vii.1998	K. Nupponen
EZHBA660–07		*Pseudochazara doerriesi*	Russia	51.717	94.4	17.vii.2000	Oleg Kosterin
EZHBA661–07		*Pseudochazara doerriesi*	Russia	51.717	94.4	17.vii.2000	Oleg Kosterin
EZHBA662–07		*Pseudochazara doerriesi*	Russia	51.717	94.4	17.vii.2000	Oleg Kosterin
EZHBA899–07		*Pseudochazara doerriesi*	Russia	51.7667	91.9333	30.vi.2004	Oleg Kosterin
EZHBA900–07		*Pseudochazara doerriesi*	Russia	51.7667	91.9333	30.vi.2004	Oleg Kosterin
EZROM089–08	HQ004207	*Chazara briseis*	Romania: Transylvania: Suatu	46.783	23.95	16.viii.2006	Dinca Vlad
EZROM848–08	HQ004205	*Chazara briseis*	Romania: Transylvania: Suatu	46.799	23.959	16.viii.2006	Dinca Vlad
EZSPM470–09	GU676107	*Pseudochazara hippolyte*	Spain: Granada: San Juan (Sierra Nevada)	37.094	-3.115	16.vii.2009	Dinca V.
EZSPN732–09	GU676410	*Pseudochazara hippolyte*	Spain: Granada: Laguna Seca, Hueneja	37.097	-2.97	18.vii.2008	S. Montagud , J. A. Garcia-Alama & J. Garcia
EZSPN733–09	GU676411	*Pseudochazara hippolyte*	Spain: Granada: Laguna Seca, Hueneja	37.097	-2.97	18.vii.2008	S. Montagud , J. A. Garcia-Alama & J. Garcia
EZSPN735–09	GU676413	*Pseudochazara hippolyte*	Spain: Granada: Laguna Seca, Hueneja	37.097	-2.97	18.vii.2008	S. Montagud , J. A. Garcia-Alama & J. Garcia
EZSPN736–09	GU676406	*Pseudochazara hippolyte*	Spain: Granada: Laguna Seca, Hueneja	37.097	-2.97	18.vii.2008	S. Montagud , J. A. Garcia-Alama & J. Garcia
EZSPN791–09	GU676354	*Pseudochazara hippolyte*	Spain: Granada: North-East Granada province	37.097	-2.97	23.vii.2008	Gil, Felipe
GWOSF831–10	JF850408	*Pseudochazara anthelea anthelea*	Cyprus	34.9559	32.9951	05.vi.2010	M. Seizmair
IRANB276–08		*Pseudochazara beroe beroe*	Iran	38.583	44.367	29.vii.2002	Vazrick Nazari
IRANB278–08		*Pseudochazara beroe beroe*	Iran	38.583	44.367	29.vii.2002	Vazrick Nazari
IRANB279–08		*Pseudochazara beroe beroe*	Iran	37.776	46.445	22.vi.2001	Vazrick Nazari
IRANB285–08		*Pseudochazara beroe aurantiaca*	Iran	36.12	51.2	16.viii.2000	Vazrick Nazari
IRANB292–08		*Pseudochazara pelopea persica*	Iran	34.603	47.055	01.vii.2001	Vazrick Nazari
LOWA019–06	FJ663351	*Chazara enervata*	Kazakhstan: Tienschan: Kurdai Pass	43.333	74.95	11.vi.2000	V.Lukhtanov
LOWA021–06	FJ663349	*Chazara enervata*	Kazakhstan: Tienschan: Kurdai Pass	43.333	74.95	11.vi.2000	V.Lukhtanov
LOWA022–06	FJ663347	*Chazara briseis magna*	Kazakhstan: Tienschan: Kurdai Pass	43.333	74.95	11.vi.2000	V.Lukhtanov
LOWA024–06	FJ664025	*Pseudochazara turkestana turkestana*	Kazakhstan: Tienschan: Kurdai Pass	43.333	74.95	11.vi.2000	V.Lukhtanov
LOWA150–06	FJ664021	*Pseudochazara hippolyte pallida*	Russia	50.1	88.417	07.vii.1999	V.Lukhtanov
LOWA315–06	FJ663353	*Chazara heydenreichi*	Kazakhstan: Ust-Kamenogorsk Region: Kendyrlik	47.5	85.183	14.vii.1997	V. Lukhtanov
LOWA316–06	FJ663352	*Chazara heydenreichi*	Kazakhstan: Ust-Kamenogorsk Region: Kendyrlik	47.5	85.183	14.vii.1997	V. Lukhtanov
LOWA516–06	FJ664024	*Pseudochazara turkestana turkestana*	Kyrgyzstan: Gultcha distr.: Chiitala	39.85	73.333	29.vii.1995	V. Lukhtanov
LOWA517–06	FJ664023	*Pseudochazara turkestana turkestana*	Kyrgyzstan: Gultcha distr.: Chiitala	39.85	73.333	29.vii.1995	V. Lukhtanov
LOWA608–06	FJ663348	*Chazara briseis maracandica*	Uzbekistan: Kashkardarinskaya obl.: Tamshush	38.967	67.4	20.vi.1994	V. Lukhtanov
LOWA680–06	FJ664020	*Pseudochazara hippolyte mercurius*	Kazakhstan: Dzhambulskaya obl.: Kurdai Pass	43.333	74.95	28.vi.1993	V. Lukhtanov
LOWA681–06	FJ664019	*Pseudochazara hippolyte mercurius*	Kazakhstan: Dzhambulskaya obl.: Kurdai Pass	43.333	74.95	28.vi.1993	V. Lukhtanov
LOWA787–06	FJ664018	*Pseudochazara hippolyte hippolyte*	Kazakhstan	47.4	83.917	22.vi.1997	V. Lukhtanov
LOWA788–06	FJ664022	*Pseudochazara turkestana tarbagata*	Kazakhstan	47.4	83.917	22.vi.1997	V. Lukhtanov
LOWAB040–07		*Pseudochazara pelopea persica*	Armenia	40.083	44.917		Andrei Sourakov
LOWAB041–07		*Pseudochazara pelopea persica*	Armenia	40.083	44.917		Andrei Sourakov
LOWAB046–07		*Pseudochazara pelopea persica*	Armenia	40.083	44.917		Andrei Sourakov
LOWAB046–07		*Pseudochazara pelopea caucasica*	Armenia	40.083	44.917		Andrei Sourakov
LOWAB047–07		*Pseudochazara pelopea persica*	Armenia	40.083	44.917		Andrei Sourakov
LOWAB048–07		*Pseudochazara pelopea persica*	Armenia	40.083	44.917		Andrei Sourakov
WMB1212–13		*Pseudochazara atlantis*	Morocco	33.025	-5.071	01.vii.2011	Vila, R., Dinca, V. & Voda, R.
WMB1213–13		*Pseudochazara atlantis*	Morocco	33.025	-5.071	01.vii.2011	Vila, R., Dinca, V. & Voda, R.
WMB2163–13		*Pseudochazara atlantis*	Morocco	31.09	-7.915	15.vii.2012	Tarrier, Michel
